# Whole-Genome Sequencing and RNA-Seq Reveal Differences in Genetic Mechanism for Flowering Response between Weedy Rice and Cultivated Rice

**DOI:** 10.3390/ijms23031608

**Published:** 2022-01-30

**Authors:** Richard S. Garcia, Sapphire Coronejo, Jonathan Concepcion, Prasanta K. Subudhi

**Affiliations:** School of Plant, Environmental, and Soil Sciences, Louisiana State University Agricultural Center, Baton Rouge, LA 70803, USA; rgarcia@agcenter.lsu.edu (R.S.G.); scoronejo@agcenter.lsu.edu (S.C.); concep10@msu.edu (J.C.)

**Keywords:** days to heading, genetic interaction, *Oryza sativa*, photosensitivity, red rice, RNA-Sequencing

## Abstract

Flowering is a key agronomic trait that influences adaptation and productivity. Previous studies have indicated the genetic complexity associated with the flowering response in a photoinsensitive weedy rice accession PSRR-1 despite the presence of a photosensitive allele of a key flowering gene *Hd1*. In this study, we used whole-genome and RNA sequencing data from both cultivated and weedy rice to add further insights. The *de novo* assembly of unaligned sequences predicted 225 genes, in which 45 were specific to PSRR-1, including two genes associated with flowering. Comparison of the variants in PSRR-1 with the 3K rice genome (RG) dataset identified unique variants within the heading date QTLs. Analyses of the RNA-Seq result under both short-day (SD) and long-day (LD) conditions revealed that many differentially expressed genes (DEGs) colocalized with the flowering QTLs, and some DEGs such as *Hd1*, *OsMADS56*, *Hd3a*, and *RFT1* had unique variants in PSRR-1. *Ehd1*, *Hd1*, *OsMADS15*, and *OsMADS56* showed different alternate splicing (AS) events between genotypes and day length conditions. *OsMADS56* was expressed in PSRR-1 but not in Cypress under both LD and SD conditions. Based on variations in both sequence and expression, the unique flowering response in PSRR-1 may be due to the high-impact variants of flowering genes, and *OsMADS56* is proposed as a key regulator for its day-neutral flowering response.

## 1. Introduction

Weedy rice (*Oryza sativa f. spontanea* Rosh), a conspecific relative of cultivated rice, is a major irritant for rice farmers in many parts of the world [1]. The most plausible hypotheses regarding its evolution from *japonica* or *indica* and/or *japonica* × *indica* hybrids have been supported by molecular studies [2,3,4,5]. The de-domestication hypothesis of weedy rice evolution is recently gaining much traction based on the whole-genome sequence (WGS) analyses [6,7,8]. There are two types of weedy rice ecotypes in the USA based on the morphology: straw-hulled (SH) weedy rice with a light, brown-colored hull; and black-hull awned (BHA) weedy rice with a black-colored hull and long awns. SH ecotypes are morphologically similar to cultivated rice, while BHA resembles wild rice *Oryza rufipogon* [9]. However, they differ in several agronomically important traits such as heading date [10]. SH weedy rice exhibited early heading compared to BHA [11].

The economic impact of weedy rice infestation in the rice growing states in southern USA is enormous due to reduced grain yield, quality, and marketability resulting from contamination with red kernels of weedy rice. As weedy rice has been noted for their persistence, researchers have focused their attention on the genetic dissection of traits such as seed shattering and seed dormancy [12,13]. Many of these adaptive features can be exploited to improve cultivated rice. Early heading is an essential weedy trait that helps them to survive and compete with cultivated rice by contributing to the escape from being harvested [14]. 

Flowering is a complex trait with wide variation among cereal crops. Investigation of the flowering mechanisms of rice as a model grass species has expanded our knowledge through its relevance to other cereal crops [15,16,17]. However, the growth and development strategies of rice as a short-day (SD) plant compromise its relevance as a model for temperate cereals such as wheat and barley, which are all long-day (LD) plants such as *Arabidopsis*. Studies on natural variation in rice have shown orthologous photoperiod pathway genes similar to *Arabidopsis* [18]. *Heading date 1* (*Hd1*), a major gene determining day length sensitivity in rice, was orthologous to *AtCO* (*CONSTANS 1*) in *Arabidopsis* [15]. Further, *Hd3a* is also an ortholog of *Arabidopsis* floral pathway integrator *Flowering Locus T* (*FT)*. *Hd1* promotes *Hd3a* expression under SD conditions and repression during LD conditions, whereas *AtCO* promotes *FT* expression under LD conditions in *Arabidopsis* [19]. Another gene, *RFT1*, a tandemly duplicated paralog of *Hd3a*, is a LD-specific florigen in rice [20]. In *Arabidopsis*, *GIGANTEA* (*GI*) plays an important role in regulating the *CO-FT* pathway [21]. However, a rice homolog *OsGI* mediates the regulation of the *Hd1-Hd3a* pathway [22]. These findings reveal that this pathway controlling photoperiodic flowering is conserved between rice and *Arabidopsis* despite their contrasting photoperiodic response. Another pathway for photoperiodic flowering in rice involves *Ehd1*, a flowering gene encoding B-type response regulator, which shows no obvious ortholog in *Arabidopsis*. *Ehd1* functions upstream of *Hd3a* and *RFT1* [23], whereas its expression is higher under LD conditions in weedy rice compared with cultivated rice [24]. In addition, *Ghd7* influences the expression levels of *Ehd1* and *Hd3a* but not *Hd1* and delays flowering by repressing their expression under LD conditions [25]. The identification of orthologous genes has been helpful for understanding flowering response pathways of both rice and *Arabidopsis*.

Analysis of the natural genetic variation in cultivated and wild germplasm at the genomic level led to an improved understanding of the evolutionary origin of weedy rice [6,7,8]. The understanding of the molecular differences of weedy and agronomic traits in cultivated and weedy rice can be useful for rice improvement [26]. The application of next-generation sequencing (NGS) facilitates the understanding of the variation in gene expression patterns through the identification of high-impact DNA polymorphisms such as single-nucleotide polymorphisms (SNPs), insertions/deletions (InDels), and structural variations (SVs) within coding regions. Furthermore, analyses of unmapped sequences provide an opportunity to examine novel genes unique to weedy rice [27].

The present study used a high-yielding long-grain rice cultivar Cypress and a SH weedy accession PSRR-1. PSRR-1 was used in a number of investigations related to seed dormancy, seed shattering, and days to heading [12,13,24]. Based on results from staggered planting experiments, both genotypes showed no response to photoperiod under both LD and SD conditions, but the hybrids between PSRR-1 and Cypress and the near-isogenic line (NIL) with introgressed PSRR-1 *Hd1* were highly photosensitive [24]. Despite no difference in the response to photoperiod between parents, six heading date QTLs were identified in recombinant inbred line populations under LD conditions [24]. Several of the known genes overlapping with QTLs were validated using introgression lines of PSRR-1 [28]. The comparison of genotyping and gene expression data of key flowering genes in the NIL and parents led us to suggest that the genetic interaction of *Hd1* with a novel genetic factor may be responsible for the photo-insensitive nature of PSRR-1 under LD conditions [24].

Based on genome-wide DNA polymorphisms, PSRR-1 was genetically closer to *indica* rice than the *japonica* rice and, therefore, its evolution from *indica* rice was more plausible [27]. Another WGS study revealed clear-cut differences in the genomic makeup between SH and BHA weedy rice, and the SH weedy rice diverged later than the BHA weedy rice [11]. In these studies, several high-impact SNPs and InDels were identified with an attempt to elucidate loci for adaptive traits such as seed dormancy and seed shattering [11,26].

In this study, we performed comparative analyses of SNPs and InDels using both whole-genome resequencing and 3K rice genome data to identify all the unique variants in SH weedy rice accession PSRR-1, in addition to analyses of SVs and unmapped sequences. We also compared the genetic variations in heading date loci and global transcriptomic profiles of PSRR-1 and cultivated rice Cypress to unravel shared and unique features for each genotype.

## 2. Results

### 2.1. Whole-Genome Sequence Analyses of PSRR-1

A total of 95,183,701 reads were generated from Cypress, while 137,685,675 reads were from PSRR-1 with assembly rates of 98% and 97%, respectively (Table S1). In total, there were 1,269,049 and 3,029,642 variants, which included SNPs, InDels, and SVs in Cypress and PSRR-1, respectively.

### 2.2. Unique SNPs and InDels in PSRR-1 through Comparison with 3K-Rice Genome Data Set

**Figure 1:**
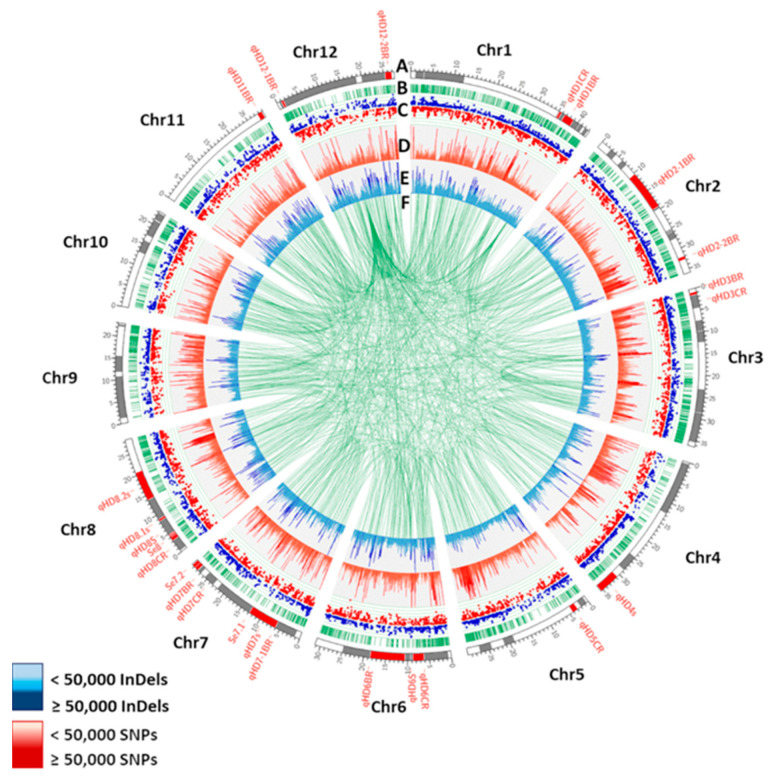
Genome-wide distributions of unique SNPs and InDels, and structural variations overlapping with DTH QTLs along with differentially expressed genes in PSRR-1. (**A**) DTH QTL segments identified from Gramene database (https://archive.gramene.org/qtl/) (accessed on 5 May 2020) [29]: grey bands, and DTH QTLs of weedy rice from literature [24,30,31,32]: red bands. (**B**) Gene distribution across chromosome (green bars). (**C**) Scatter plot of differentially expressed genes (DEGs). Blue dots and red dots represent upregulated and downregulated DEGs (|log2 fold change| > 1.5 and p-adj < 0.05) in all combined pair-wise conditions, respectively. (**D**) Unique SNP density of PSRR-1 versus 3K Rice Genome (RG) dataset at 10-kb window (red bars). (**E**) Unique InDel density of PSRR-1 versus 3K RG dataset at 10-kb window. (**F**) Structural variations in PSRR-1 (green lines).

**Figure 2:**
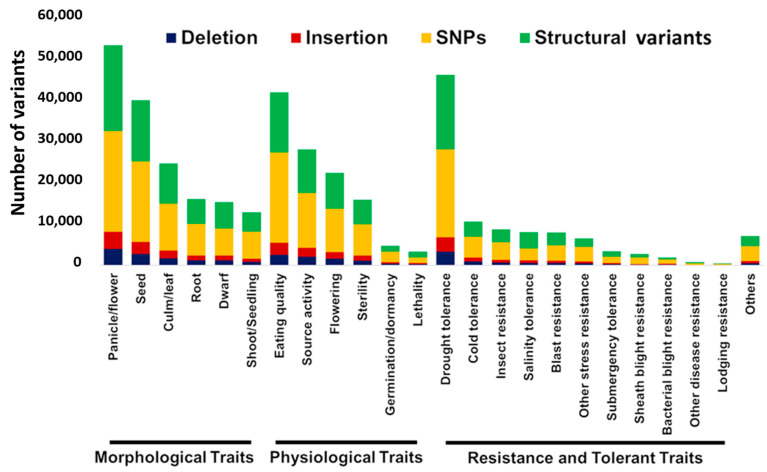
Unique high-impact SNPs, InDels, and structural variants (SVs) associated with QTLs for agronomically important traits mined from Q-TARO database (http://qtaro.abr.affrc.go.jp/) (accessed on 28 October 2020) [33]. Blue, red, yellow, and green bars correspond to deletion, insertion, SNPs, and SVs, respectively.

A comparison of 2,190,324 homozygous SNPs from PSRR-1 with 4,817,964 SNPs from the 3K RG-filtered SNP dataset revealed 1,485,635 common-to-low-frequency SNP alleles, 200,582 rare SNP alleles, and 504,107 unique alleles. Common-to-low-frequency and rare alleles were PSRR-1 SNPs that were called in the 3K RG SNP dataset with minor allele frequencies (MAFs) of ≥0.01 and <0.01, respectively. The SNPs that were not called in the 3K RG SNP dataset were considered unique to PSRR-1. On the other hand, there were 413,374 unique InDels in PSRR-1. The distribution of the SNPs and InDels was spread across all chromosomes (Figure 1). These unique SNP and InDels were categorized according to the predicted variant effects. Those with high-impact were considered due to their high probability of generating gain or loss-of-function and/or changes in the expression levels of the desired genes (Figure S1). In addition, structural variants (SVs) in PSRR-1 showed an unbiased distribution across all chromosomes (Figure S2). The SVs and unique variants showed several high-impact mutations within the agronomically important QTLs (Figure 2), which encompassed morphological, physiological, and stress tolerance traits. The panicle/flowering traits had the greatest number of variants. The days to heading (DTH) QTLs alone harbored several unique mutations (Figure 1, Table S2).

### 2.3. Unmapped Sequence of PSRR-1

Unmapped reads of PSRR-1 were assembled using rice as a reference, and there were 225 predicted genes of which only 180 genes were annotated with significant matches (Table S3). The remaining 45 predicted sequences had a very low to non-significant hits and were most likely unique to PSRR-1. The E-value was used as the main parameter for significance followed by bit score and percent identity. Twenty six of the 180 genes with E-value 0.0 were considered redundant and were therefore more likely identical, while 154 genes were closely related and/or almost identical with E-value < 1.0 × 10^−10^. From this set of significant sequence matches, 13, 31, and 136 were annotated as uncharacterized, characterized, and hypothetical proteins, respectively. Both uncharacterized and hypothetical proteins had not been experimentally validated. The genes were homologous to cultivated rice, wild rice, weedy rice, and other grass sequences. Moreover, a closer look at the protein families and domains of the predicted genes revealed that the majority of protein families belong to DISEASE RESISTANCE PROTEIN RP (PTHR23155), P-loop containing nucleoside triphosphate hydrolase (IPR027417), NB-ARC (IPR002182), Rx, N-terminal (IPR041118), and Leucine-rich repeat (IPR001611) (Table S4).

### 2.4. Transcriptome Analysis of PSRR-1 and Cypress under Short and Long-Day Conditions

**Figure 3:**
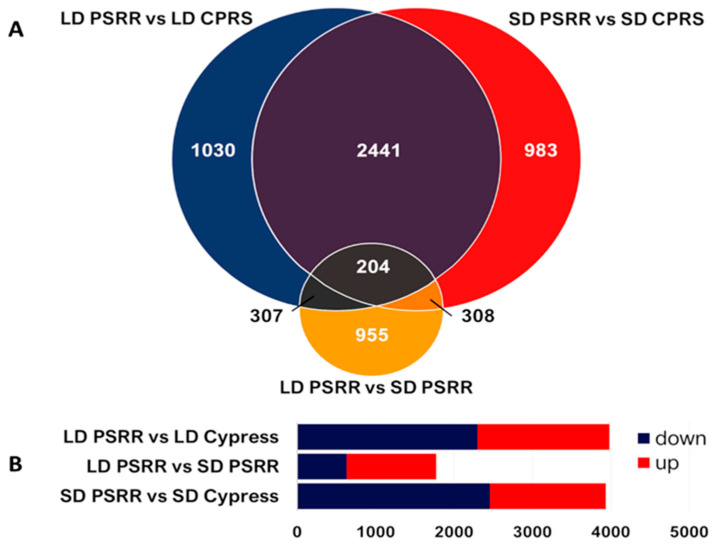
Differentially expressed gene (DEG) profiles of PSRR-1 and Cypress under different day length conditions. (**A**) Venn diagram showing overlapping of DEGs between PSRR-1 and Cypress (CPRS) under long-day (LD) and short-day (SD) conditions with pair-wise comparisons: LD PSRR vs. LD CPRS, LD PSRR vs. SD CPRS, and SD PSRR vs. SD CPRS (|log2 fold change| ≥ 1.5 and *padj* < 0.05). (**B**) Bar graphs showing number of DEGs from different pair-wise comparisons along with the regulation pattern.

RNA sequencing of leaf tissues from PSRR-1 and Cypress under long-day and short-day conditions resulted in 316 and 330 million reads, respectively (Table S5). The mean assembly for both genotypes was 96%. Differentially expressed genes (DEGs) were identified for each pairwise comparison between Cypress and PSRR under different conditions. Each pairwise comparison included the genotype-specific DEGs for LD and SD conditions (LD PSRR vs. LD Cypress and SD PSRR vs. SD Cypress, respectively) and day length-specific DEGs (LD PSRR vs. SD PSRR). The total number of DEGs was 3982 (1683 upregulated and 2299 downregulated), 3936 (1476 upregulated and 2460 downregulated), and 1774 (1142 upregulated and 632 downregulated) in LD PSRR vs. LD Cypress, SD PSRR vs. SD Cypress, and LD PSRR vs. SD PSRR, respectively (Figure 3, Table S6).

### 2.5. Gene Ontology (GO) and Pathway Analysis of DEGs under SD and LD Conditions

In agriGO analysis, 78, 59, and 80 significant GO terms were identified in LD PSRR vs. LD Cypress, SD PSRR vs. SD Cypress, and LD PSRR vs. SD PSRR, respectively (Figure S3). Plant Reactome pathway analyses of DEGs showed major plant pathways involved in cellular, growth, developmental processes, metabolism and regulation, and responses to abiotic and biotic stimuli and stresses (Table 1). Both GO and pathway results indicated involvement in significant biological functions encompassing a diverse range of plant processes including reproduction. The pathways involved in flowering networking and control could be reflected from the notable number of DEGs in the ‘reproductive structure development’ and ‘hormone signaling, transport, and metabolism’ pathways. The DEGs within these categories were involved in flowering. For example, LD and SD-regulated florigens were commonly found under all conditions under ‘reproductive structure development’ such as *Hd3a* and *RFT1*. On the other hand, the ‘hormone signaling, transport, and metabolism’ pathway had DEGs involved in gibberellin and auxin signaling, which are important in flowering responses. Additionally, several of these DEGs belong to the transcription factor (TF) families that are largely involved in flowering such as CO-like and MIKC-MADS TFs (Figure S4).

### 2.6. DEGs and Variants in Flowering Genes and Flowering-Related Pathway

**Figure 4:**
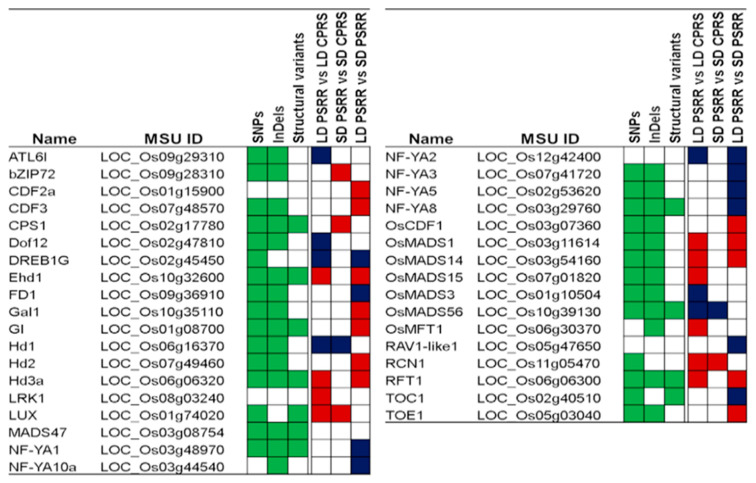
Flowering genes with unique SNP, InDels, and SVs, and the regulation pattern under different day length conditions. Green-filled boxes indicate variants within the gene. Blue and red-filled boxes indicate the regulation pattern (up- and downregulated, respectively) (|log2 fold change| ≥ 1.5 and *padj* < 0.05) in pairwise comparisons of day length conditions between long day (LD) and short day (SD) of PSRR-1 (PSRR) and Cypress (CPRS).

Among the DEGs, there were 35 genes related to flowering under all day length conditions (Figure 4). Some of these were major rice flowering genes such as *Hd1*, *Hd3a*, *RFT1*, and *Ehd1*, while some indirectly involved in the flowering response were NF-YA and OsMADS-type flowering genes. These DEGs were constantly regulated under both LD and SD conditions, and the expression level varied due to genotype under some instances. For example, *Hd1* and *OsMADS56* of PSRR-1 showed upregulation regardless of day length conditions. Similarly, *LUX* and *RCN1* showed downregulation under all day length conditions. There were DEGs that were only regulated on specific day lengths (day length effect) such as *Ehd1, RFT1*, and *Hd3a*, which were downregulated under LD conditions. Furthermore, the differentially expressed flowering-related genes revealed unique SNPs, InDels, and SVs (Figure 4, Tables S7 and S8). For example, *Hd1*, *OsMADS56*, *Ehd1*, *Hd3a*, and *RFT1* had unique SNPs and InDels in PSRR-1 compared with Cypress.

### 2.7. Differentially Expressed Flowering-Related Genes Overlapping DTH QTLs

**Figure 5:**
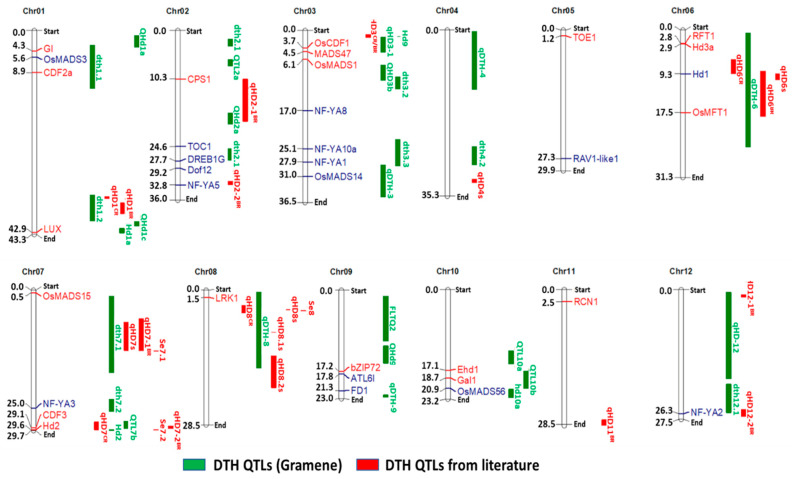
Overlapping of flowering-related genes with days to heading (DTH) QTLs. Upregulated and downregulated genes are shown in blue and red fonts, respectively. Vertical green bars represent published DTH QTLs from Gramene (https://archive.gramene.org/qtl/) (accessed on 5 May 2020) [29] and vertical red bars represent QTLs from earlier studies involving weedy rice [24,30,31,32].

Twenty of the 35 differentially expressed flowering-related genes identified from the RNA-Seq analysis (Figure 4) overlapped with DTH QTLs from previous studies involving weedy rice (Figure 5). Some of the DEGs belonged to the major flowering genes such as *RFT1* and *Hd3a*, which overlapped with *qDTH-6*. *Hd1* overlapped with *qDTH-6* and *qHD6^BR^*, while some flowering genes such as *GI*, *OsMADS14*, and *OsMADS56* overlapped with *dth1.1*, *qDTH-3*, and *qQTL10a*, respectively.

### 2.8. Alternative Splicing Events of Flowering Genes under Short- and Long-Day Conditions

A total of 49,707 alternative splicing (AS) events were identified in PSRR-1 (25,179) and Cypress (24,528) (Table S7). The AS profiles of selected flowering genes revealed no differences between genotypes and day length conditions in *Ghd7*, *Hd3a*, *RFT1*, and *OsMADS14* (Table 2). However, *Hd1* and *OsMADS56* had AS profile differences between PSRR-1 and Cypress but not within LD and SD conditions. Further, *Ehd1* showed no AS event differences between different day lengths in Cypress, while PSRR-1 exhibited AS events only under SD conditions. *Ehd1*, *Hd1*, and *OsMADS56* showed different AS events between genotype or day length conditions, which indicated altered transcript profiles despite similar flowering patterns of PSRR-1 and Cypress.

### 2.9. qRT-PCR Validation of Flowering Genes

**Figure 6:**
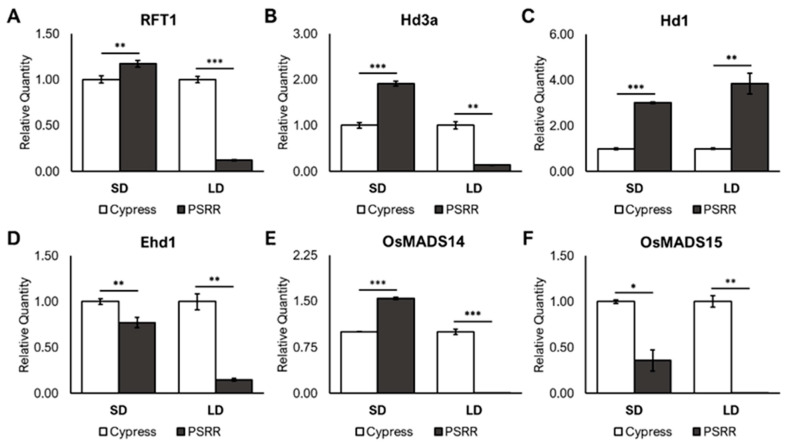
Relative expression levels of flowering-related genes in Cypress and PSRR-1 under short-day (SD) and long-day (LD) conditions. (**A**–**F**) Real-time quantitative reverse transcription PCR results of flowering genes using Ubq5 as the internal control and Cypress as the reference sample. Bar graph depicts mean relative quantity ± standard deviation. Asterisk (*) indicates significance by two-tailed Student’s t-test assuming unequal variances; * *p* < 0.05, ** *p* < 0.01, and *** *p* < 0.001.

The qRT-PCR analysis of selected flowering genes showed similar expression profiles with RNA-Seq of Cypress and PSRR-1 under LD conditions; however, the trend was different under SD conditions (Figure 6). Under LD conditions, *RFT1*, *Hd3a*, *Ehd1*, *OsMADS14*, and *OsMADS15* were downregulated and *Hd1* showed upregulation in PSRR-1. On the other hand, *RFT1*, *Hd3a*, *Hd1*, and *OsMADS14* were upregulated, and *Ehd1* and *OsMADS15* were downregulated under SD conditions in PSRR-1. Both *Ehd1* and *OsMADS15* did not reflect the expression pattern in qRT-PCR as with RNA-seq (Figure 4), which could be due to their elimination in RNA-seq analysis because of the threshold setting (|log2 fold change| ≥ 1.5 and *padj* < 0.05).

**Figure 7:**
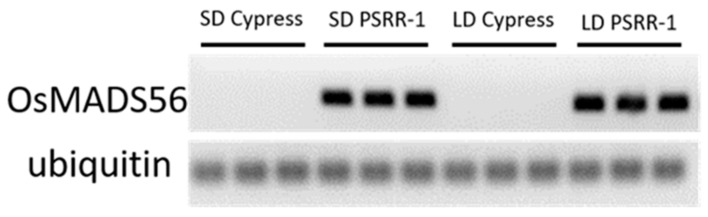
Semiquantitative RT-PCR amplification of *OsMADS56* in Cypress and PSRR-1 under short-day (SD) and long-day (LD) conditions.

In addition, *OsMADS56* (*LOC_Os10g39130*) showed amplification in PSRR-1 but not in Cypress (Figure 7). The lack of expression under LD or SD conditions in Cypress validated the RNA-Seq results in which BAM files showed no *OsMADS56* reads. Analysis of WGS data showed a large deletion (1008 bp at position 20,863,490–20,864,497) in Cypress, resulting in a loss of the start codon in *OsMADS56* (Figures S5 and S6).

## 3. Discussions

Flowering time is a key agronomic attribute that determines local adaptation and productivity in crop plants. Understanding the genetic basis of flowering was helpful because the development of varieties with uniform flowering assists in harvesting and helping to remove or reduce the response to photoperiod for expanding the adaptation and cultivation of crops to larger geographic regions. Flowering time is equally important for weedy species because it enhances their competitiveness and persistence. While early flowering allows the shedding of seeds and development of a seedbank before crop harvest, the overlapping of flowering time between weedy rice and crops ensures harvesting along with the crop and increases the chance of gene flow between weeds and crop species [17]. Genetic studies on flowering in US weedy rice have been limited to QTL mapping [24,31,32,34]. Demonstrations of the tremendous amount of hidden variation for flowering time in weedy rice have indicated the usefulness of weedy rice as a model to investigate the genes and their interaction controlling this important agronomic trait [24]. Therefore, the present investigation was carried out to provide some insights into the molecular genetic basis of flowering response in a straw hull weedy rice accession through comparison with cultivated rice using whole-genome resequencing and global transcriptomics approaches.

The de novo assembly of unmapped reads of PSRR-1 revealed novel genes as with earlier studies in rice [27]. The majority of these genes were homologous to the *indica* rice group (Table S3), confirming earlier studies regarding the origin of straw-hulled weedy rice from *indica* rice [6,26,31,32]. Among the predicted genes, a few had protein structure domains similar to heading date/flowering genes. For example, *g146.t1* contig had an RNA-binding domain (IPR035979) of *OsRRMh* (*LOC_Os09g34070*) and RNA-recognition motif (RRM) of *OsFCA* (*LOC_Os09g03610*). These two genes, *OsRRMh* and *OsFCA*, were associated with the transition from the vegetative to reproductive stage [35] and the autonomous flowering pathway in rice, respectively [36]. *OsRRMh* was also associated with increased yield [37], confirming the finding of Liu and Cai [35] who reported longer panicles in RNAi lines and reduced fertility and spikelet number in overexpressing lines compared with wild type. Studies on the ab initio gene prediction such as this help in the discovery of ‘novel’ genes. Although in vitro validation such as the PCR experiment was not performed, the likelihood of identifying new genes is not impossible, as illustrated in ab initio gene predictions in rats, which was confirmed through PCR validation [38]. The above genes with protein families similar to flowering-related genes suggest their involvement in flowering time variation in weedy rice.

Despite being the closest relative of cultivated rice, weedy rice exhibits wide genetic diversity and complex genetic background [39]. Although de-domestication events involving a few genes were recently hypothesized for the origin of US weedy rice [6], further investigation is needed to understand the reversion at these loci to the *indica* type, which are not cultivated in the US [40]. The hidden variation of flowering time genes in the weedy rice accession with day-neutral response could be a useful model to trace the origin of weedy rice through analysis of this domesticated trait [24].

PSRR-1 had numerous unique InDels and SNPs when compared against the 3K RG dataset, comprising several subspecies of cultivated rice and wild rice. Based on the comparison of SNPs and InDels, more variants have been reported between PSRR-1 and a *japonica* rice genotype compared to an *indica* genotype [26]. These unique variants were co-localized in the heading date QTLs reported earlier [31,32]. Similarly, heading date QTLs from our earlier study [24] involving PSRR-1 overlapped with the unique variants in *qHD1^CR^*, *qHD3^CR^*, *qHD5^CR^, qHD6^CR^, qHD7^CR^*, and *qHD8^CR^*. The presence of these unique variants in PSRR-1 could have arisen from spontaneous mutation during the de-domestication process. Pre-existing variations, new mutations, and variations contributed greatly to the adaptation of weedy rice [7]. Weedy rice may not have simply reverted to domesticated forms but may have employed different sets of genes for improving its adaptation depending on the selection pressure in a particular environment. For example, in addition to other adaptive genes such as *sh4*, *sh-h*, and *Rc*, flowering genes, *OsMADS51* and *Ehd4* were associated with the de-domestication process [7]. The presence of these unique variants, specifically the high-impact mutations and SVs, should be useful for functional genomic studies not only related to flowering but also with other agronomically important traits.

Among the DEGs identified from the comparative transcriptomics approach involving PSRR-1 and Cypress under SD and LD conditions, many overlapped with heading date QTLs and were related to flowering based on the literature (Figure 4 and Figure 5). Apart from unique variants and SVs identified in the flowering genes such as *Hd3a*, *RFT1*, *OsMADS14*, *OsMADS15*, and *OsMADS56* (Tables S8 and S9), there were changes in the splicing events (Table 2 and Table S7), which could have contributed to the differential expression pattern between PSRR-1 and Cypress. Differential AS events in rice were reported as coping mechanisms under adverse environments [41,42,43], and specifically, the flowering time in coconut was influenced by AS of the FT gene [44].

**Figure 8:**
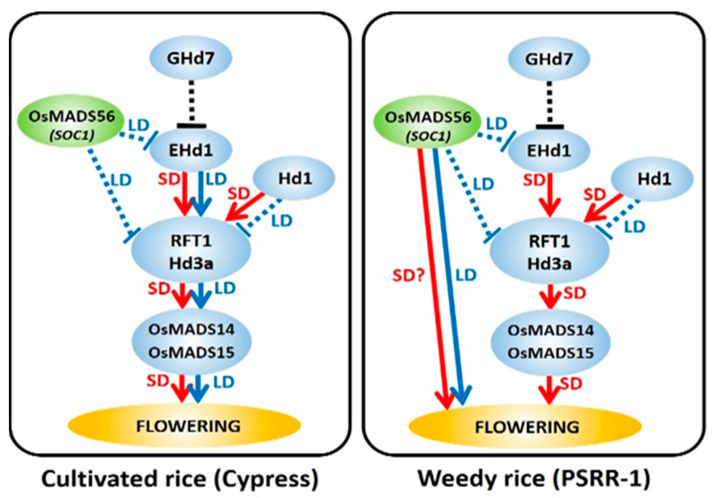
A simplified model for flowering in cultivated rice (Cypress) and weedy rice (PSRR-1) under long-day (LD: blue arrow lines) and short-day (SD: red arrow lines) conditions. In Cypress, *RFT1/Hd3a* signals downstream genes (*OsMADS14* and *OsMADS15*) to induce flowering. Under LD and SD, *Hd1* and *Ehd1* regulate *RFT1/Hd3a*, while *OsMADS56* acts as an upstream regulator of *Ehd1*, *RFT1*, and *Hd3a*. In weedy rice, *OsMADS56* is the major flowering activator under LD, while *RFT1/Hd3a* switches back to control flowering during SD. The role of *OsMADS56* during SD is still unclear, indicated by ‘?’.

In this study, PSRR-1 did not show any delay in flowering under LD conditions despite the downregulation of *RFT1*, *Hd3a*, *Ehd1*, and the downstream genes such as *OsMADS14* and *OsMADS15*. This observation suggests a different mechanism involving genes other than *Hd1* or *Ehd1*. Based on the results from sequence variation, gene expression, and AS events, *OsMADS56* (LOC_Os10g39130) was considered as a potential candidate for regulating flowering time in PSRR-1 under LD conditions. Both *OsMADS56* and its closely related *OsMADS50* (LOC_Os03g03070) are orthologous to *SOC1* (*SUPPRESSOR OF OVEREXPRESSION OF CONSTANS1*) of *Arabidopsis* (*AtSOC1*) [45] and regulate flowering-related genes downstream of *CO* [46], independent of the *Hd1* pathway. However, only *OsMADS56* showed a genotype-specific AS event and was upregulated in PSRR-1 compared with Cypress under both LD and SD conditions. Both encode MIKC-type MADS-box proteins [47]. While *OsMADS50* is a LD-specific flowering activator [48,49], *OsMADS56* functions as a negative regulator under LD conditions [50,51]. In *Arabidopsis*, *AtSOC1* acts downstream of *CO* and *FT* that eventually initiates flowering via the activation of *AtLFY* and *AtAP1* [18]. In contrast, *OsMADS56* operates upstream of flowering gene activators, *Ehd1*, *RFT1*, and *Hd3a*, which were downregulated in *OsMADS56*-overexpressing rice plants, leading to delayed flowering under LD conditions [52], unlike PSRR-1 in this study. Therefore, the early flowering or day-neutral response in PSRR-1 under LD conditions could be due to the increased expression of *OsMADS56* despite the downregulation of *Ehd1*, *RFT1*, *Hd3a*, *OsMADS14*, and *OsMADS15* and upregulation of *Hd1*. However, under SD conditions, *OsMADS56*, which is still upregulated, did not suppress the expression of *RFT1*, *Hd3a*, and *OsMADS14*. Considering the above results, we hypothesize that *OsMADS56* promoted flowering by acting directly in a similar fashion as *AtSOC1* under LD conditions (Figure 8). The function of *OsMADS56* was reversed in a similar manner as *Hd1*, which promotes flowering in rice under SD conditions [15] in contrast to CO promoting flowering under LD in *Arabidopsis* [53]. However, under SD conditions, *OsMADS56* had no effect in PSRR-1-like *AtCO* in *Arabidopsis*, in which all major flowering genes reverted to their normal expression mode. As all major flowering genes were active under SD conditions, PSRR-1 likely switched back to its normal flowering mechanism. However, the role of *OsMADS56* under SD conditions could not be assessed, because it was also upregulated under those conditions.

This study demonstrated an approach involving both transcriptomics and WGS to analyze unique variants, expression patterns, and AS events in flowering genes, leading to the discovery of a novel flowering mechanism involving *OsMADS56* unique to weedy rice accession PSRR-1. However, further investigation is needed to gain insights into the role of *OsMADS56* and its interaction in flowering time regulation.

## 4. Materials and Methods

### 4.1. Plant Materials and Cultivation

Plant materials used in this study included SH weedy rice accession ‘PSRR-1’ and a rice cultivar ‘Cypress’. ‘Cypress’ is a high-yielding long-grain rice cultivar developed from the ‘L-202’/‘Lemont’ cross at the Louisiana Agricultural Experiment Station [54], and ‘PSRR-1′ is a straw-hulled US weedy rice [12]. Both PSRR-1 and Cypress are photo-insensitive or day-neutral [24]. Planting was performed under greenhouse conditions at the LSU Agricultural Center in Baton Rouge, LA (30°24′41.7″ N, 91°10′21.8″ W) around mid-April and mid-July for exposure to long- and short-day conditions, respectively.

### 4.2. Whole-Genome Sequencing and Analyses

The Illumina FASTQ files of PSRR-1(PRJNA413818) and Cypress (PRJNA598851) were retrieved from the sequence read archive at the National Center for Biotechnology Information (NCBI). Burrows-Wheeler Aligner (BWA; MEM-algorithm; version 0.7.8) [55,56] was used for the mapping of high-quality filtered reads against the rice reference genome (Os-Nipponbare-Reference-IRGSP-1.0), downloaded from the Rice Genome Annotation Project [57]. Genomic variant identification was performed using the Genome Analysis Toolkit (GATK) version 4.2.3 [58], while structural variants (SV) were called using Lumpy version 0.3.1 [59]. Genome-wide coverage was estimated using SAMtools version 1.9 [54]. SnpEff version 4.3 [60] was used to predict the variant effects of SNPs, InDels, and SVs. The genome-wide distribution of DNA polymorphisms was analyzed by calculating their frequency at 10-kb intervals on each rice chromosome. R version 3.6.1 was used to visualize the distribution of SNPs and InDels on rice chromosomes. Nucleotide sequences were extracted for alignment with the consensus sequence from BAM files using IGV version 2.8.4 [61] and FastaAlternateReferenceMaker under GATK tools. Nucleotide sequences were then translated into proteins using ExPASy [62]. Alignment was performed with Jalview version 2.11.1.4 [63] using MUSCLE [64].

### 4.3. De Novo Assembly and Analyses of Unmapped Sequences

The unmapped reads of the PSRR-1 genome were assembled using the assembler, MaSuRCA (v3.2.1), with default options [65]. RepeatMasker (v4.0.6) [66] with default options was used to mask the assembled genome using rice as a model followed by gene prediction via Augustus v3.2.1 [67]. The annotations of predicted genes were performed using the NCBI-BLAST+ (2.11.0) [68] and UniProt [69] against plant databases. Additionally, the protein domain and family were identified based on the functional annotation by InterProScan (5.47–82.0) [70].

### 4.4. Comparison SNPs and InDels with 3K Rice Genome

The dataset containing 4.8 million filtered SNPs from the 3K RG was downloaded from the Rice SNP-Seek database (https://snp-seek.irri.org/) (accessed on 5 October 2020) [71]. This dataset comprised all biallelic and homozygous SNPs, which were filtered using parameters of alternative/minor allele frequency (MAF) of at least 0.01 (which covers common to low-frequency variation) and missing call proportions of at most 0.2. The SNPs were compared with PSRR-1 SNPs to enumerate the common SNPs and identify the SNPs unique to PSRR-1. The InDel dataset was downloaded from the Rice SNP-Seek database and was compared with PSRR-1 InDels. The InDels that were not called based on InDel position from the 3K RG were designated as unique InDels. From this unique dataset, the nonsynonymous and high-impact mutations that specifically caused rare amino acid variants, splice acceptor/donor variants, stop lost/gained, and start lost, were identified [60].

### 4.5. Agronomic Trait-Related Variants

Unique SNPs, InDels, and SVs from QTLs associated with morphological and physiological attributes were identified by co-localizing the position using the Q-TARO database (http://qtaro.abr.affrc.go.jp/) (accessed on 28 October 2020) [33]. The nonsynonymous and high-impact variants were used in the QTL co-localization to illustrate the impacts of unique alleles and SVs in PSRR-1 as a potential genomic resource.

### 4.6. Flowering Genes and Its Gene Signaling Network

The unique SNPs and InDels lying within the flowering genes and/or gene signaling network related to flowering between PSRR-1 and Cypress (Table S8) and structural variations with reference to Nipponbare (Table S9) were identified. The set of genes was initially obtained from the rice flowering pathway database at WikiPathways (Table S10) [72]. Subsequently, the information on differentially expressed flowering genes was used to infer the regulation pattern observed between PSRR-1 and Cypress.

### 4.7. RNA-Sequencing

Two sets of both Cypress and PSRR-1 were cultivated for exposure to short-day (mid-April planting) and long-day conditions (mid-July planting). These were grown under greenhouse conditions at Louisiana State University Campus Greenhouse in Baton Rouge, Louisiana (30°24′41.7″ N, 91°10′21.8″ W) with the day/night temperature set at 24 °C/13 °C, respectively. Leaf tissues from the penultimate fully expanded leaves were collected at the booting stage (~53 days after sowing). Samples were taken from three biological replicates and were stored in a −80 °C freezer until RNA extraction.

Total RNA was extracted using TRIzol (Invitrogen, CA, USA) based on the manufacturer’s protocol. The quality and quantity of the extracted RNA were assessed using the ND-1000 Spectrophotometer (Thermofisher Scientific, Waltham, MA, USA) and Agilent 2100 Bioanalyzer (Agilent Technologies, Santa Clara, CA, USA). DNAse treatment was performed using PerfeCTa DNase I (QuantaBio, Beverly, MA, USA) following the manufacturer’s instructions. Purified RNA samples were submitted to Novogene Corporation Inc. (Sacramento, CA, USA) for cDNA library construction and 150-bp paired-end sequencing using the Illumina Novaseq platform.

The quality of the raw reads was checked with FastQC (Babraham Bioinformatics, https://www.bioinformatics.babraham.ac.uk/) (accessed on 15 September 2021), wherein reads with a Phred quality score ≥30 were used for downstream analysis. The processed paired-end reads were mapped to the rice reference genome (Os-Nipponbare-Reference-IRGSP-1.0 downloaded from the Rice Genome Annotation Project [56,57] using HISAT2 version 2.0.1 [73]. The resulting mapped reads were processed using featureCounts [74], and differentially expressed genes (DEGs) were determined from the raw count table using the DESeq2 R package [75]. To identify the DEGs, a Benjamini–Hochberg adjusted *p*-value of <0.05 and log2 fold change ≥ 1.5 were set as criteria. Gene ontology was performed on the DEGs to determine the biological significance with respect to the biological processes (BPs), molecular functions (MFs), and cellular localization (CC) of their proteins using singular enrichment analysis with AgriGO v2 [76]. Plant pathway analyses were performed using Plant Reactome to identify the participation of DEGs in plant pathways affected under different conditions [77], and transcription factors were identified and classified using the PlantTFDB v5.0 database [78].

### 4.8. Co-localization of Flowering-Related DEGs in Heading/Flowering Date QTLs

The flowering-related DEGs identified from PSRR-1 and Cypress under both day length conditions were co-localized with published days to heading (DTH) QTLs from the Gramene QTL database (https://archive.gramene.org/qtl/) (accessed on 5 May 2020) [29] and from previous publications in weedy rice [24,31,32]. In the case of weedy-related DTH, only those QTLs linked in straw hull weedy rice mapping populations were used for co-localization. Co-localized DEGs for each QTL were illustrated using MapChart 2.3 [79].

### 4.9. Alternative Splicing of Flowering-Related DEGs

Transcripts for each sample mapped by HISAT2 were assembled via StringTie v.1.3.3 using the default settings [80]. The transcript assemblies were combined for each genotype per treatment through StringTie using the merge option. Subsequently, the resulting GTF files were processed using the AStalavista web server (accessed on 4 January 2022) [81] for alternative splicing (AS) event identification. The evaluation of these AS events was performed following the method of Sammeth et al. [82], which identifies events such as intron retention (IR), exon skipping (ES), alternative 3′ splice sites (A3SS), alternative 5′ splice sites (A5SS), mutually exclusive exon (MXE), and complex events (i.e., A5SS + A3SS, A5SS + ES + A3SS, and the like).

### 4.10. Validation of Gene Expression via qRT-PCR

To validate the gene expression patterns of selected DEGs from the RNA-seq analysis, 1.0 μg of DNase-treated RNA was used to synthesize cDNA with the iScript cDNA Synthesis Kit (Bio-Rad Laboratories, Inc., Hercules, CA, USA). The consensus sequence from the RNA-seq and published transcript sequences (Phytozome) were used for designing primers. PrimerQuest Tool (Integrated DNA Technologies, Inc., Coralville, IA, USA) was used for the primer design (Table S11) and primers were synthesized from Integrated DNA Technologies, Inc., Coralville, IA, USA.

Expression analysis of the flowering genes was performed in 96-well plates on an Applied Biosystems QuantStudio 3 Real-Time PCR system using iTaqTM Universal SYBR Green supermix (Bio-Rad Laboratories, Inc., Hercules, CA, USA). The total reaction volume was 10 μL containing the following components: 5.0 µL of 2× iTaqTM universal SYBR^®^ Green supermix, 0.3 µL of 5 µM of each primer, 1.0 µL of 1:10 cDNA, and 3.4 µL of sterilized distilled water. PCR cycle conditions were as follows: 5 min of initial denaturation at 95 °C, 40 cycles of real-time PCR with 2-step amplification consisting of 95 °C for 15 s and 60 °C for 1 min, and a 3-step melting curve comprising 95 °C for 15 s, 60 °C for 1 min, and 95 °C for 15 s with a rate of 1.6 °C/s for each step. Reactions with no cDNA were used as a negative control. The expression level of the target genes and a reference gene (*UBQ5*) was measured in three biological replicates per tissue using gene-specific primers. Expression levels for genes were determined using the 2^−ΔΔCT^ method [83].

## Figures and Tables

**Table 1:** Distribution (%) of differentially expressed genes on plant pathway nodes in PSRR-1 and Cypress under short- and long-day conditions.

Plant Pathway		LD PSRR vs. LD CPRS	SD PSRR vs. SD CPRS	LD PSRR vs. SD PSRR
Cellular processes	Protein metabolism: translation	5.0	3.7	1.1
	Cell cycle	3.5	3.3	1.9
	Cellular processes	2.3	1.8	0.6
	DNA replication: activation of the pre-replicative complex	0.6	0.4	0.0
Circadian rhythm	Circadian rhythm	0.0	0.0	0.6
Growth and developmental processes	Reproductive structure development	11.7	7.2	11.5
	Growth and developmental processes	4.2	2.7	4.7
	Vegetative structure development	1.5	0.6	2.1
	Amine and polyamine biosynthesis	1.3	0.0	0.8
Metabolism and regulation	Metabolism and regulation	17.9	20.8	19.2
	Secondary metabolism	11.9	13.0	14.9
	Hormone signaling, transport, and metabolism	10.0	11.8	13.4
	Amino acid metabolism	12.5	10.8	8.2
	Carbohydrate metabolism	5.0	8.0	6.6
	Cofactor biosyntheses	5.0	3.7	4.0
	Inorganic nutrients metabolism	2.1	4.5	2.1
	Fatty acid and lipid metabolism	2.1	2.7	2.1
	Detoxification	0.0	1.2	1.3
	Amine and polyamine biosynthesis	0.0	0.6	0.4
	Nucleotide metabolism	0.0	0.4	0.0
	Photorespiration	0.2	0.2	0.0
Responses to stimuli: abiotic stimuli and stresses	Responses to stimuli: abiotic stimuli and stresses	1.0	1.0	1.5
	Response to cold temperature	0.4	0.4	0.8
	Response to drought	0.8	0.4	0.4
	Response to heavy metals	0.4	0.4	0.8
	Response to submergence	0.0	0.4	0.0
	Response to salinity	0.2	0.0	0.2
Responses to stimuli: biotic stimuli and stresses	Responses to stimuli: biotic stimuli and stresses	0.2	0.0	0.4
	Recognition of fungal and bacterial pathogens and immunity response	0.2	0.0	0.4

SD, Short-day; LD, Long-day.

**Table 2:** Alternative splicing events of differentially expressed major flowering genes.

Gene Symbol	SD PSRR	LD PSRR	SD Cypress	LD Cypress
Ehd1	A5SS	-	A5SS	A5SS
Ghd7	-	-	-	-
Hd1	-	-	A3SS	A3SS
Hd3a	-	-	-	-
OsMADS14	A3SS	A3SS	A3SS	A3SS
OsMADS15	-	-	-	A3SS, ES, IR, IR1 + IR2
OsMADS56	Complex IR	Complex IR	-	-
RFT1	-	-	-	-

IR, Intron retention; ES, exon skipping; A3SS, alternative 3′ splice sites; A5SS, alternative 5′ splice sites; MXE, mutually exclusive exon; Complex, A5SS + A3SS, A5SS + ES + A3SS, and the like.

## Data Availability

The data presented in this study are available in the article and supplementary material. All RNA-Sequencing data are openly available in the sequence read archive (SRA) of NCBI under the accession number PRJNA801867.

## Supplementary Materials

The following supporting information can be downloaded at: https://www.mdpi.com/article/10.3390/ijms23031608/s1.
