# Supplementary material for: Whole-Genome Sequencing and RNA-Seq Reveal Differences in Genetic Mechanism for Flowering Response between Weedy Rice and Cultivated Rice
Source: Int J Mol Sci. 2022 Jan 30;23(3):1608. doi: 10.3390/ijms23031608 (PMC8836195; doi:10.3390/ijms23031608)
Supplement: Supplementary file 1 [file ijms-23-01608-s001.zip › Supplementary Figures S1-S6.pdf]

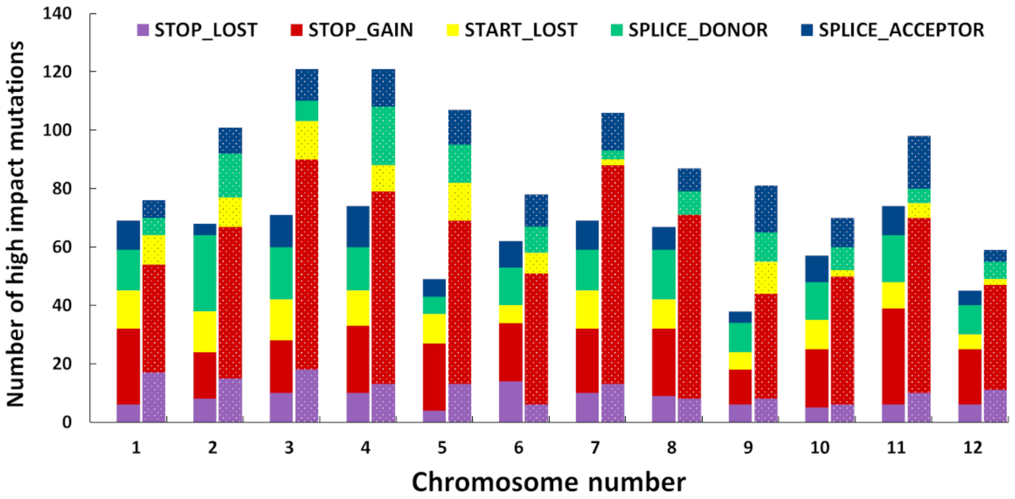

Figure S1. Unique SNP and InDels with high impact identified in PSRR-1 through comparison with 3K-Rice genome data set.

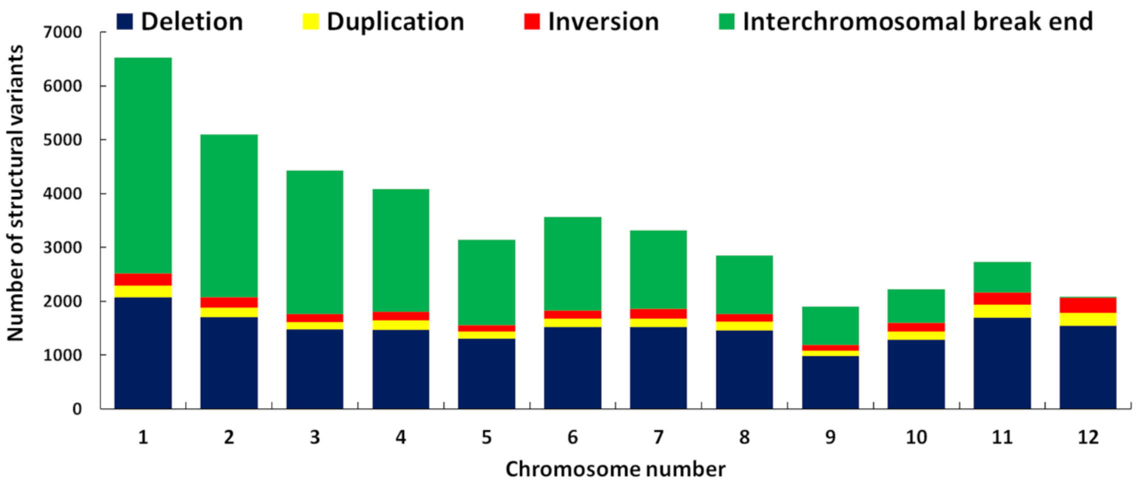

Figure S2. Types of structural variants identified in PSRR-1 through comparison with 3K-Rice genome data set and their distribution in rice genome

A

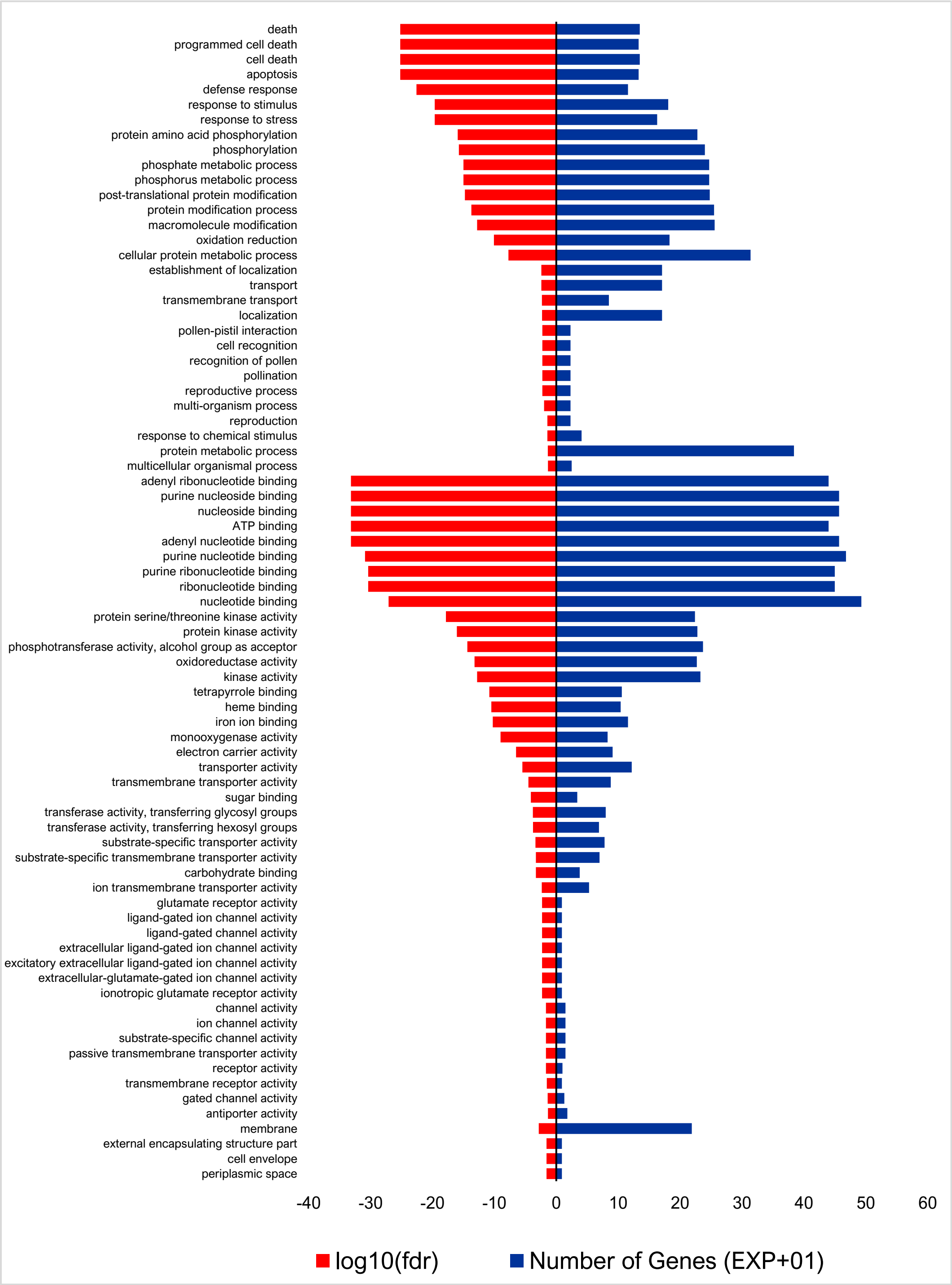
death
programmed cell death
cell death
apoptosis
defense response
response to stimulus
response to stress
protein amino acid phosphorylation
phosphorylation
phosphate metabolic process
phosphorus metabolic process
post-translational protein modification
protein modification process
macromolecule modification
oxidation reduction
cellular protein metabolic process
establishment of localization
transport
transmembrane transport
localization
pollen-pistil interaction
cell recognition
recognition of pollen
pollination
reproductive process
multi-organism process
reproduction
response to chemical stimulus
protein metabolic process
multicellular organismal process
adenyl ribonucleotide binding
purine nucleoside binding
nucleoside binding
ATP binding
adenyl nucleotide binding
purine nucleotide binding
purine ribonucleotide binding
ribonucleotide binding
nucleotide binding
protein serine/threonine kinase activity
protein kinase activity
phosphotransferase activity, alcohol group as acceptor
oxidoreductase activity
kinase activity
tetrapyrrole binding
heme binding
iron ion binding
monooxygenase activity
electron carrier activity
transporter activity
transmembrane transporter activity
sugar binding
transferase activity, transferring glycosyl groups
transferase activity, transferring hexosyl groups
substrate-specific transporter activity
substrate-specific transmembrane transporter activity
carbohydrate binding
ion transmembrane transporter activity
glutamate receptor activity
ligand-gated ion channel activity
ligand-gated channel activity
extracellular ligand-gated ion channel activity
excitatory extracellular ligand-gated ion channel activity
extracellular-glutamate-gated ion channel activity
ionotropic glutamate receptor activity
channel activity
ion channel activity
substrate-specific channel activity
passive transmembrane transporter activity
receptor activity
transmembrane receptor activity
gated channel activity
antiporter activity
membrane
external encapsulating structure part
cell envelope
periplasmic space
-40 -30 -20 -10 0 10 20 30 40 50 60
log10(fdr)
Number of Genes (EXP+01)

B

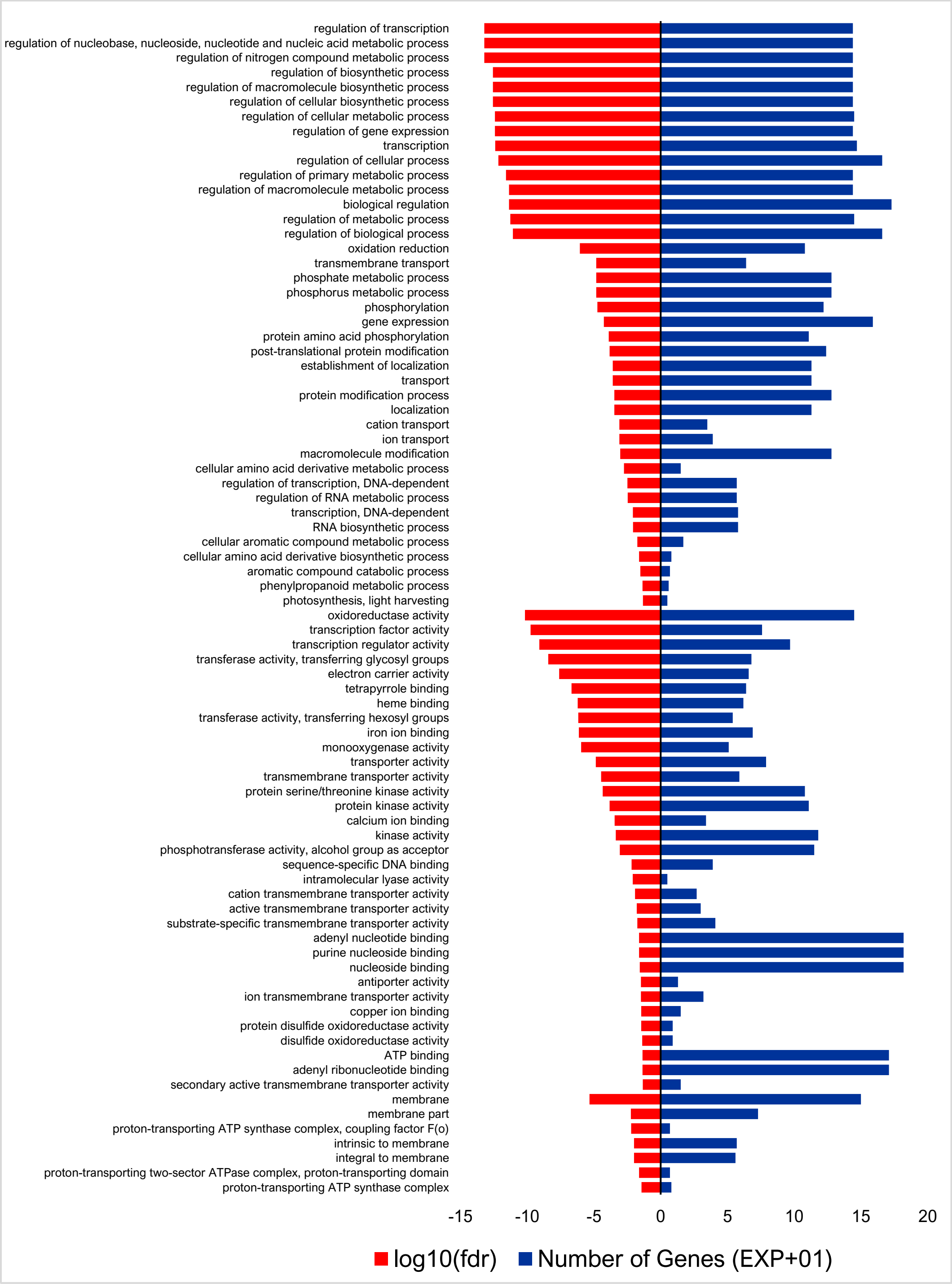

regulation of transcription
regulation of nucleobase, nucleoside, nucleotide and nucleic acid metabolic process
regulation of nitrogen compound metabolic process
regulation of biosynthetic process
regulation of macromolecule biosynthetic process
regulation of cellular biosynthetic process
regulation of cellular metabolic process
regulation of gene expression
transcription
regulation of cellular process
regulation of primary metabolic process
regulation of macromolecule metabolic process
biological regulation
regulation of metabolic process
regulation of biological process
oxidation reduction
transmembrane transport
phosphate metabolic process
phosphorus metabolic process
phosphorylation
gene expression
protein amino acid phosphorylation
post-translational protein modification
establishment of localization
transport
protein modification process
localization
cation transport
ion transport
macromolecule modification
cellular amino acid derivative metabolic process
regulation of transcription, DNA-dependent
regulation of RNA metabolic process
transcription, DNA-dependent
RNA biosynthetic process
cellular aromatic compound metabolic process
cellular amino acid derivative biosynthetic process
aromatic compound catabolic process
phenylpropanoid metabolic process
photosynthesis, light harvesting
oxidoreductase activity
transcription factor activity
transcription regulator activity
transferase activity, transferring glycosyl groups
electron carrier activity
tetrapyrrole binding
heme binding
transferase activity, transferring hexosyl groups
iron ion binding
monooxygenase activity
transporter activity
transmembrane transporter activity
protein serine/threonine kinase activity
protein kinase activity
calcium ion binding
kinase activity
phosphotransferase activity, alcohol group as acceptor
sequence-specific DNA binding
intramolecular lyase activity
cation transmembrane transporter activity
active transmembrane transporter activity
substrate-specific transmembrane transporter activity
adenyl nucleotide binding
purine nucleoside binding
nucleoside binding
antiporter activity
ion transmembrane transporter activity
copper ion binding
protein disulfide oxidoreductase activity
disulfide oxidoreductase activity
ATP binding
adenyl ribonucleotide binding
secondary active transmembrane transporter activity
membrane
membrane part
proton-transporting ATP synthase complex, coupling factor F(o)
intrinsic to membrane
integral to membrane
proton-transporting two-sector ATPase complex, proton-transporting domain
proton-transporting ATP synthase complex
-15
-10
-5
0
5
10
15
20
log10(fdr)
Number of Genes (EXP+01)

C

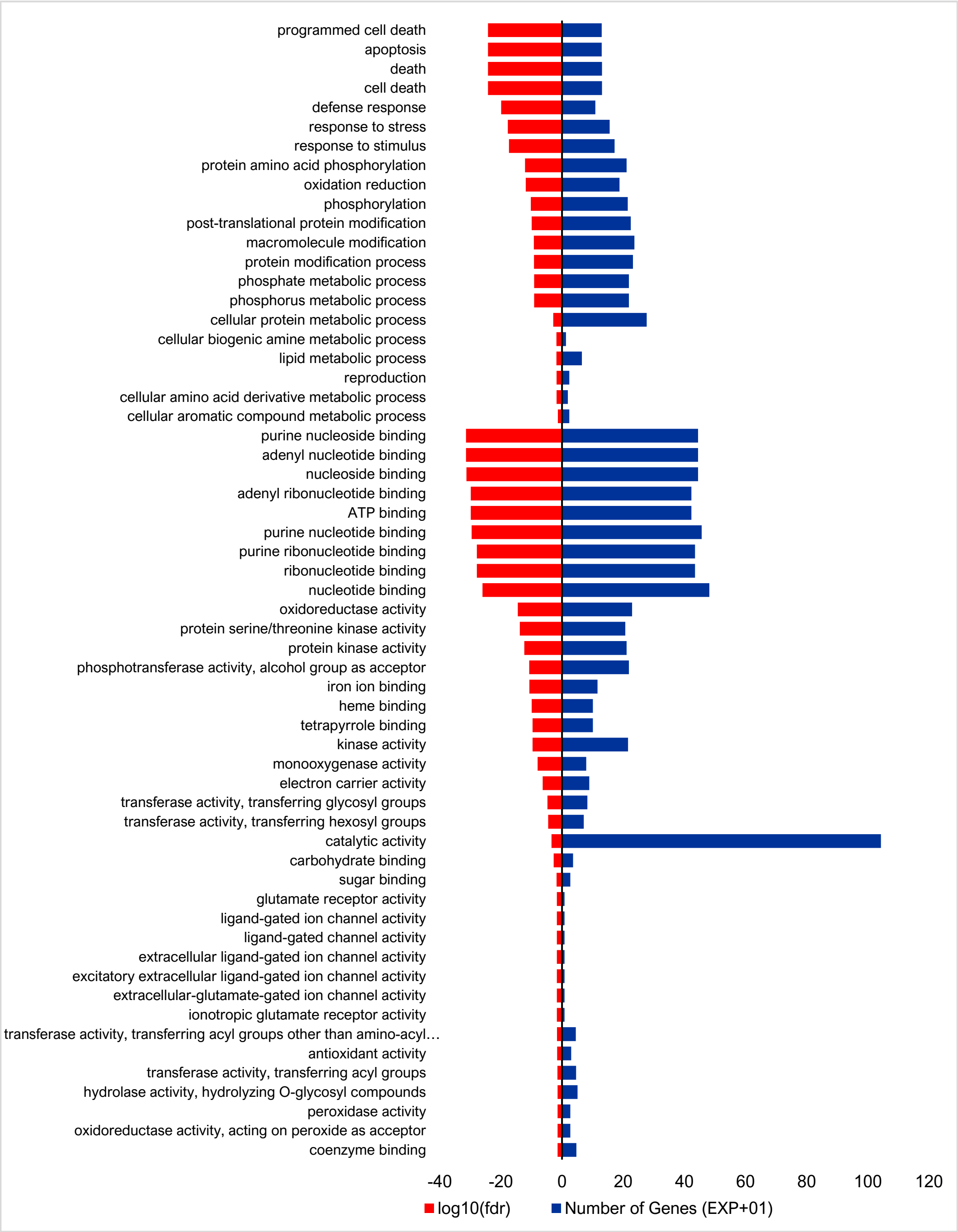

Figure S3. Gene ontology of differentially expressed genes between PSRR-1 and Cypress under different daylength conditions. (A) Long day PSRR-1 vs long day Cypress, (B) long day PSRR-1 vs short day PSRR-1, and (C) short day PSRR-1 vs short day Cypress.

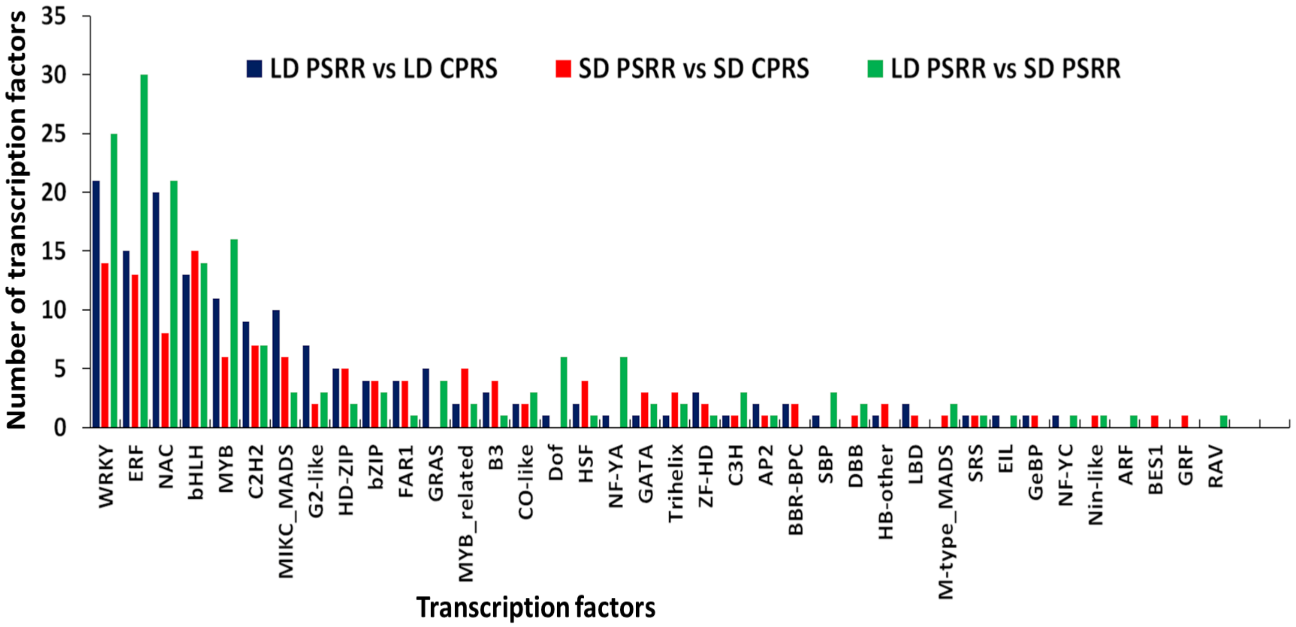

Figure S4. List of transcription factor families and their frequencies represented among the differentially expressed genes under different daylength conditions. (A) Long-day PSRR-1 vs long-day Cypress, (B) long-day PSRR-1 vs short-day PSRR-1, and (C) short-day PSRR-1 vs short-day Cypress.

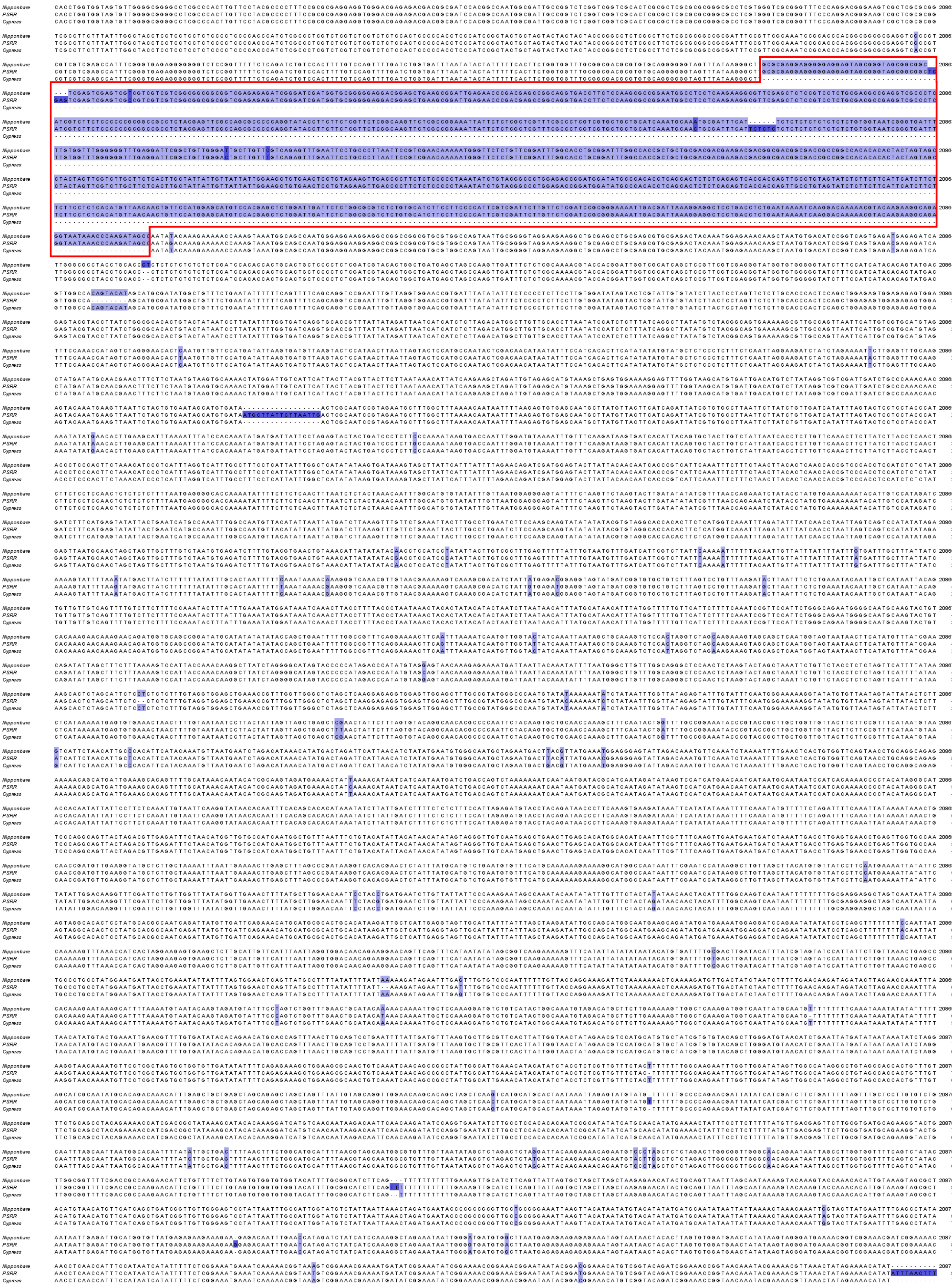
Nipponbare
PSRR
Cypress

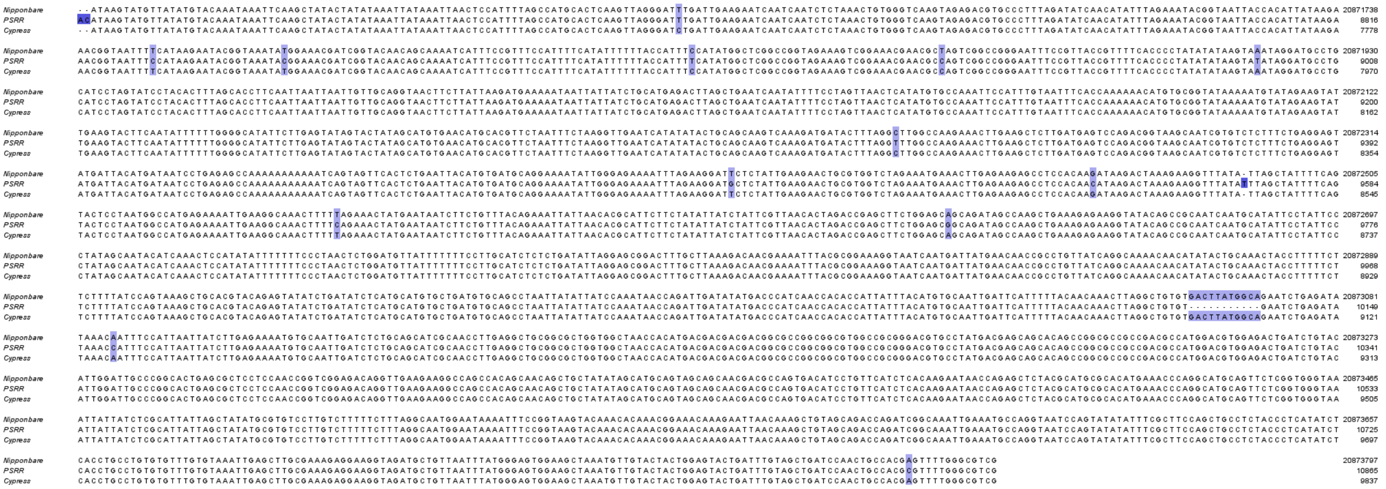

Figure S5. Nucleotide sequence alignment of *OsMADS56* (*LOC_Os10g39130*) in Nipponbare, Cypress, PSRR-1. The sequences of OsMADS56 were aligned with MUSCLE using Jalview (version 2.11.1.4). Numbers on the right side of the sequences are base pair coordinates in reference to Nipponbare. Dark blue shade indicates differences in nucleotide sequence in all three samples while light blue signifies variation in one sample. Dash represents gaps in the sequence. Red box highlights the 1008 bp deletion in Cypress.

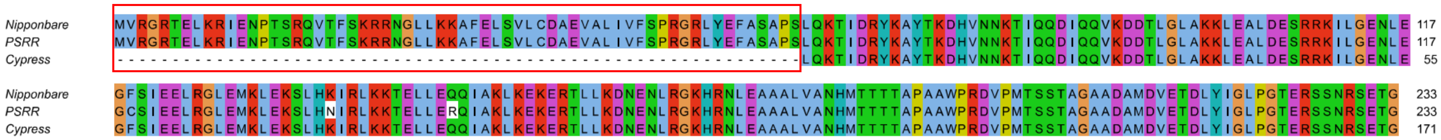

Figure S6. Protein sequence alignment of *OsMADS56* (LOC_Os10g39130.1) in Nipponbare, Cypress, PSRR-1. The sequences of OsMADS56 were aligned with MUSCLE using Jalview (version 2.11.1.4). Color shade signifies amino acid profile used in ClustalX alignments. Dash symbolizes gaps in the sequence. Red box highlights absence of start codon due to the 1008 bp deletion involving the CDS region in Cypress.
